# Precise and efficient insertion of A673T mutation in APP gene using MSYM

**DOI:** 10.1016/j.gendis.2023.101154

**Published:** 2023-10-27

**Authors:** Qing Xia, Zijie Liu, Xinyu Yang, Jiaying Xiao, Xue Zhao, Yu Zhao, Feifei Zheng, Fangliang Ge, Ke Ye, Lulu Liu, Dayong Wang, Xu Gao

**Affiliations:** a Department of Biochemistry and Molecular Biology, Harbin Medical University, Harbin, Heilongjiang 150000, China; b Dongzhimen Hospital, Beijing University of Chinese Medicine, Beijing 100700, China; c Basic Medical Institute, Heilongjiang Medical Science Academy, Harbin, Heilongjiang 150000, China; d Translational Medicine Center of Northern China, Harbin, Heilongjiang 150000, China; e Key Laboratory of Heilongjiang Province for Genetically Modified Animals, Harbin Medical University, Harbin, Heilongjiang 150000, China

Alzheimer's disease (AD) is a progressive neurodegenerative disorder causing memory loss, cognitive decline, language impairment, and disorientation, which impose an enormous burden on caregivers and the public health sector. A673T as a protective mutation has great therapeutic potential in AD.^1^^,^^2^ Therefore, a combination of stem cell therapy and A673T mutation existing in natural people based on gene targeting techniques such as CRISPR-Cas9 have been suggested as promising and exciting new developments. Homology-directed repair (HDR), relatively rare in mammalian cells, is necessary to generate a specific sequence change such as point mutations.^3^ Efficient HDR and inefficient non-homologous DNA end joining are needed.^4^^,^^5^ Moreover, the HDR pathway repairs double-strand breaks via homologous recombination; a synthesized single-stranded oligodeoxynucleotide (ssODN) with a homologous sequence as donor templates is simultaneously introduced, thereby enabling intended sequence changes.^5^ However, obstacles remain. HDR pathway disrupted by indels arises from subsequent re-editing in previously edited loci. The undesirable re-editing will recut the target loci until they are sufficiently modified to prevent further detection (Fig. 1A).

**Figure 1:**
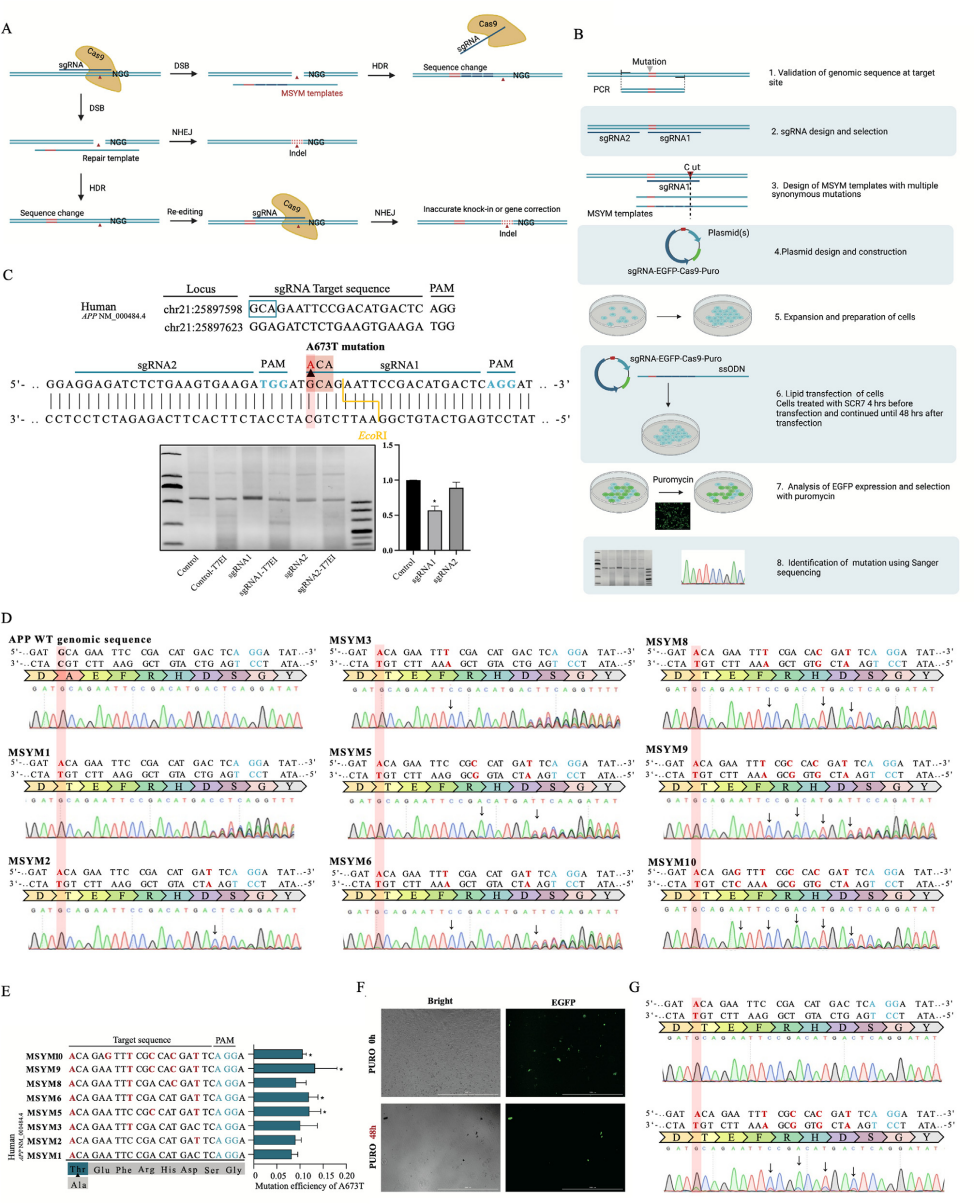
Precise and efficient insertion of A673T mutation in APP gene using MSYM. **(A)** MSYM, a scarless gene editing strategy, was devised to overcome undesirable re-editing of a previously edited locus by the CRISPR/Cas9 complex in the HDR pathway for the introduction of specific mutations with high efficiency and accuracy. **(B)** Overview of the MSYM workflow. **(C)** sgRNAs were functionally tested in HEK293T cells using the T7E1 assay. **(D)** Sanger sequencing was used to verify the A673T editing efficiency of different MSYM design schemes. **(E)** The mutation efficiency of A673T in HEK293T cells using MSYM. **(F)** MSYM9 template and MSYM1 template were co-transfected with sgRNA1-pCas9-EGFP plasmid (1:1) into iPS cells separately. **(G)** Sanger sequencing was used to verify the A673T editing efficiency in iPS cells using MSYM. All data are presented as mean ± standard error of the mean. ^∗^*P* < 0.05, ^∗∗^*P* < 0.01, ^∗∗∗^*P* < 0.001 *vs*. control. Figures in this manuscript were created with BioRender.com.

Based on this, we developed a CRISPR/Cas9-based scarless genome-editing approach termed “MSYM”, in the hope of blocking re-editing and inducing A673T mutation in iPS cells with high efficiency and accuracy. Reprogrammed iPS cells can be used to explore AD mechanisms or screen for transplanting them into the hypothalamus because of possible useful therapies. More importantly, about half of the known human pathogenic genetic variants are point mutations. The difficulty of point mutation correcting by single-base editing is improving the efficiency of HDR-mediated gene editing. Therefore, we hope MSYM could be exploited as a supplementary method to provide more options for researchers and clinicians in gene therapy.

MSYM, a scarless gene editing strategy, we devised to overcome undesirable re-editing of a previously edited locus by the CRISPR/Cas9 complex in the HDR pathway for the introduction of specific mutations and with high efficiency and accuracy. In detail, MSYM is constructing multiple synonymous mutations in ssODN templates according to the central dogma of molecular biology, to induce silent mutations in the PAM and/or in the guide RNA target sequence, which makes the previously edited locus sufficiently modified to prevent further detection and re-editing (Fig. 1A).

We have outlined the general MSYM workflow from sgRNA design to sequencing in Figure 1B. Below we discuss important considerations for specific parts of the MSYM protocol. Notably, we chose to induce multiple synonymous mutations to reduce unnecessary amino acid alteration according to the central dogma of molecular biology. Synonymous mutations can specify the same amino acid, which does not cause any phenotype. However, synonymous mutations were not found at the PAM site in this study, which does not affect the introduction of synonymous mutation at the PAM site as a feasible approach for increasing the accuracy of HDR-mediated gene editing in other studies. Moreover, we chose to induce a different number of synonymous mutations at the site that resides as close as possible to the selected cleavage site based on the efficient CRISPR-Cas9 “knock-in” strategy. We observed that HDR-mediated editing accuracy increased concordantly with the number of synonymous mutations and established a method termed “MSYM” for scarless genome editing. HEK293T cells were used to validate the feasibility of MSYM.

In addition, studies have shown that ssODN with short homologous arms produces more indels than donor templates with long homologous arms. Therefore, we chose the homology arms of ssODN larger than 50 bp in our studies. We also chose to use a Cas9/gRNA vector carrying EGFP and Puro as the backbone so that after analysis of EGFP expression and selection with puromycin, targeting efficiency would be further improved. Most critically, as an alternative DNA repair pathway that competes with the HDR pathway, the non-homologous DNA end-joining pathway can be inhibited by SCR7 targeting DNA ligase IV. Therefore, we treated cells with SCR7 4 h before transfection and continued until 48 h after transfection to promote the HDR pathway at the expense of the non-homologous DNA end-joining pathway. Taken together, our studies apply multiple steps to achieve the increased accuracy of HDR-mediated gene editing.

To test the validity and feasibility of MSYM, we first selected the most effective APP-targeting sgRNA (sgRNA1 or sgRNA2, http://crispr.mit.edu/, Table S1) for Cas endonuclease using a T7E1 assay in HEK293T cells. Robust cleavage activity was evident in the groups transfected with the Cas endonuclease together with sgRNA1 (Fig. 1C). According to the target sequence of gRNA1, we next generated all possible sequences of gRNA and DNA with synonymous mutations (WT or WT with A673T) and explored the binding activity between gRNA and DNA using CRISPR-off pipeline. A higher CRISPR-off score indicates a higher binding tendency between gRNA and DNA. We found that the number of synonymous mutations on DNA commonly negatively correlated with binding tendency between gRNA and WT (Fig. S1A) or A673T (Fig. S1E) synonymous target sequences. Exceptions were also observed in DNA with WT (Fig. S1B) or A673T (Fig. S1F), indicating the influence of the position and type of synonymous mutation on the binding tendency do exist. We also explored the influence on the binding activity between gRNA and target sequence if the synonymous mutations were on gRNA1 (Figs. S1C, D, G, H and Table S2–5). Similar results showed the number of synonymous mutations on gRNA commonly negatively correlated with binding tendency between gRNA with WT (Fig. S1C) or A673T (Fig. S1G) and DNA target sequences. Exceptions were also observed due to the position and type of synonymous mutation in gRNA with WT (Fig. S1D) or A673T (Fig. S1H). These observations suggested that MSYM may be a feasible strategy to overcome undesirable re-editing by affecting the binding activity between gRNA and DNA.

According to the sgRNA1 target sequence, we devised ten ssODN templates (MSYM templates, Table S3) with A673T mutation and/or multiple synonymous mutations at different loci around the cut site (Fig. S2A). Here, we focus on the number of synonymous mutations and the distance of cut-to-mutation. According to the target sequence of MSYM templates, we predicted the binding activity between gRNA and DNA. Binding activities showed a positive correlation with the number of synonymous mutations on gRNA or DNA and a positive correlation with the distance of cut-to-mutation (Fig. S2B, C). After that, we constructed ten vectors containing “MSYM” sequences, PAM, and mcherry. MSYM sequences were designed according to the sequence of MSYM templates (Fig. S3A). sgRNA1 was co-transfected into HEK293T cells at a ratio of 1:3 with these vectors separately (“MSYM” T1 to “MSYM” T10). Immunofluorescence assay showed that the expression level of mCherry in HEK293T cells transfected with “MSYM” T1 was lower than that in HEK293T cells transfected with “MSYM” T2 and other “MSYM” targets (Fig. S3B, D), which was consistent with the WB results (Fig. S3C, E). The expression level of mCherry in HEK293T cells gradually increased with the increase of the number of synonymous mutations in “MSYM” sequences, that is, the cutting efficiency decreased (Fig. S3B, C). Furthermore, we designed ten “MSYM” sgRNAs according to the sequence of MSYM templates (Fig. S4A). “MSYM” sgRNAs-pCas9-EGFP were transfected into HEK293T cells separately (“MSYM” S1 to “MSYM” S10). Immunofluorescence assay showed that the expression level of APP in HEK293T cells transfected with “MSYM” S1 was lower than that in HEK293T cells transfected with “MSYM” S8 and “MSYM” S9 (Fig. S4B, C), which were similar to the WB results (Fig. S3D, E). The expression level of APP in HEK293T cells gradually increased with the increase of the number of synonymous mutations in “MSYM” sgRNAs, that is, the cutting efficiency decreased (Fig. S3B, D). These observations suggested that MSYM may reduce the re-editing.

To develop high-efficiency MSYM design schemes for inducing A673T mutation in iPS cells with high efficiency and accuracy, we used HEK293T cells to co-transfect the MSYM templates (MSYM1, MSYM2, MSYM3, MSYM5, MSYM6, MSYM8, MSYM9, MSYM10) with sgRNA1-pCas9-EGFP plasmid (1:1) and added Scr7 inhibitor to promote HDR before transfection. The culture medium containing puromycin was replaced 6–8 h after transfection, and DNA was extracted at 72 h after transfection. The target fragment near the mutation site of APP A673T was amplified by PCR, and then the A673T mutation results were analyzed by Sanger sequencing. As shown in Figure 1D, the mutation efficiency of A673T in HEK293T cells gradually increased with the increase of the number of synonymous mutations in MSYM templates, that is, the re-editing efficiency decreased. Specially, MSYM9 showed the best result (Fig. 1E).

We used the MSYM9 template and MSYM1 template to co-transfect with sgRNA1-pCas9-EGFP plasmid (1:1) into iPS cells separately and added Scr7 inhibitor to promote HDR before transfection. The culture medium containing puromycin was replaced 6–8 h after transfection, and the target fragment near the mutation site of APP A673T was amplified by PCR (Fig. 1F). Sanger sequencing confirmed that the mutation efficiency of A673T in iPS cells transfected with MSYM9 template was higher than that in iPS cells transfected with MSYM1 template (Fig. 1G). We induced A673T mutation in iPS cells with high efficiency and accuracy using MSYM, which is a scarless editing protocol. Furthermore, the related information of potential off-target sites (http://crispr.mit.edu/) was listed in Figure S5. Sequence alignment showed that these potential off-target sites were not in the coding region.

## Author contributions

D.W. and X.G. conceived and supervised the research, and revised the manuscript for critical review. Q.X., Z.L., and X.Y. performed the overall study and drafted the manuscript. Q.X., J.X., X.Z., Y.Z., and F.Z. conducted experiments and/or data analysis. F.G. and K.Y. contributed to data collection and participated in data analysis. All authors read and approved the final version of the manuscript.

## Conflict of interests

The authors have declared that no competing interests exist.

## Funding

This work was supported by the 10.13039/501100001809National Natural Science Foundation of China (No. 81701078), the 10.13039/501100005046Natural Science Foundation of Heilongjiang Province of China (Outstanding Youth Foundation, No. YQ2022H003), 10.13039/501100002858China Postdoctoral Science Foundation (No. 2016M600261, 2018T110317), Heilongjiang Postdoctoral Financial Assistance (China) (No. LBH-Z15163), and Heilongjiang Touyan Innovation Team Program (China).

## Figures and Tables

**Figure:**
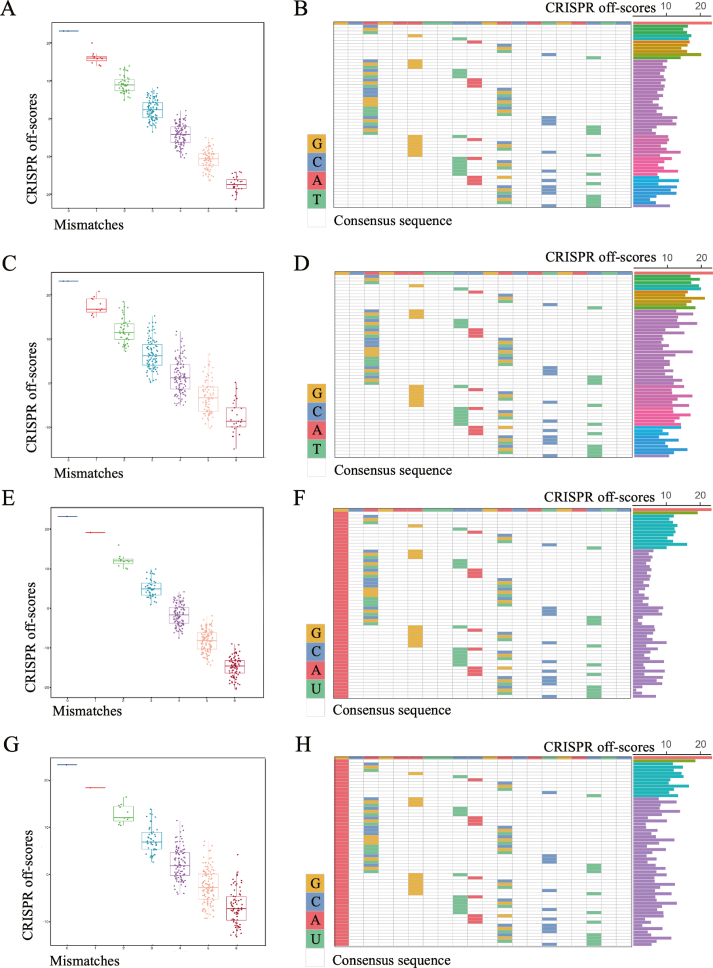
Figure S1 Predicted binding activity between gRNA and DNA with synonymous mutations according to CRISPR-off scores. **(A, B)** The binding activity between gRNA and DNA with synonymous mutations. **(C, D)** The binding activity between gRNA with synonymous mutations and DNA. **(E, F)** The binding activity between gRNA and DNA with synonymous mutations and A673T mutation. **(G, H)** The binding activity between gRNA with synonymous mutations and A673T mutation and DNA. The X-axis shows the number of mutations, and the Y-axis shows CRISPR-off scores. The left panel shows the sequences with synonymous mutations, and the right panel shows the bar plot of CRISPR-off scores.

**Figure:**
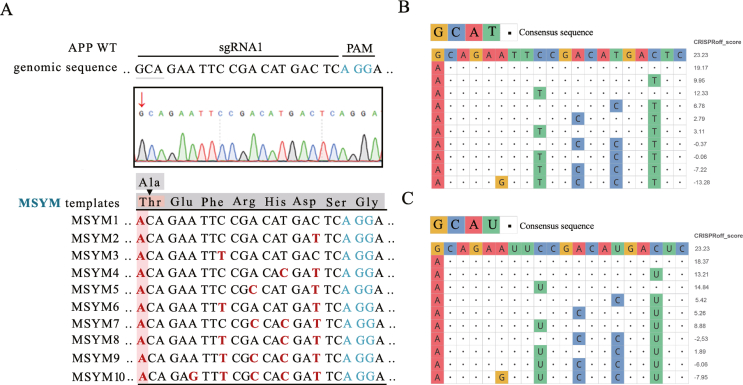
Figure S2 Predicted re-editing efficiency of MSYM. **(A)** Ten ssODN templates (MSYM templates) with A673T mutation (red) and multiple synonymous mutations (red) at different loci around the cut site are designed to test the validity and feasibility of MSYM. **(B)** The binding activity between gRNA and DNA with synonymous mutations. **(C)** The binding activity between gRNA with synonymous mutations and DNA. The left panel shows the sequences with synonymous mutations, and the right panel shows the bar plot of CRISPR-off scores. ssODN, synthesized single-stranded oligodeoxynucleotides.

**Figure:**
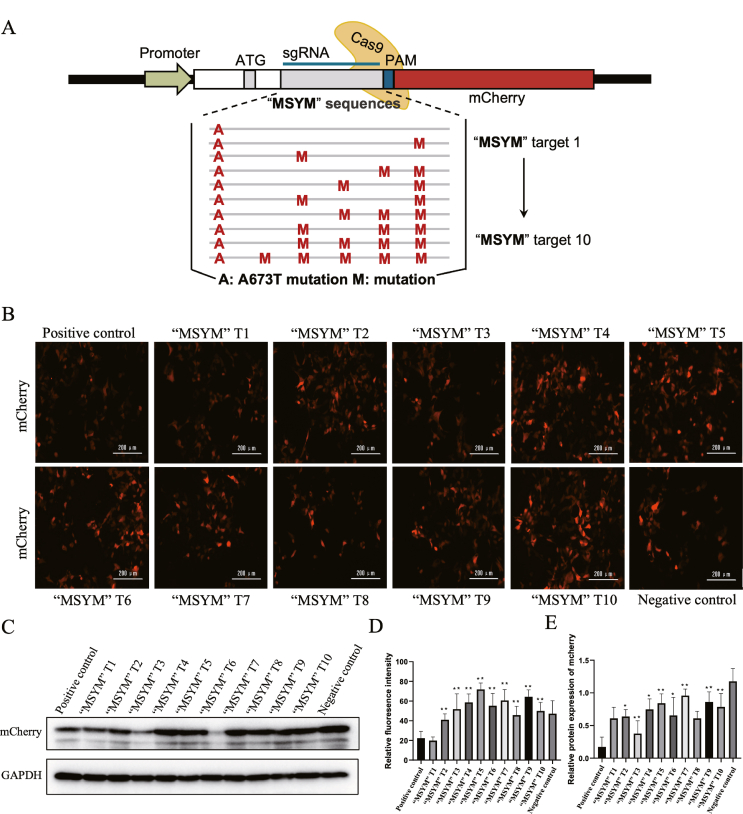
Figure S3 The cutting efficiency test using gRNA1 targeting “MSYM” targets. **(A)** Vectors containing “MSYM” sequences, PAM, and mCherry. MSYM sequences were designed according to the sequence of MSYM templates. **(B, D)** HEK293T cells transfected with “MSYM” targets and gRNA1 were labeled with anti-mCherry antibodies (red). Scale bars: 200 μm. **(C, E)** Protein levels of mCherry in HEK293T cells transfected with “MSYM” targets and gRNA1-pCas9-EGFP were detected by western blot analysis. All data are presented as mean ± standard error of the mean. ∗*P* < 0.05, ∗∗*P* < 0.01, ∗∗∗*P* < 0.001 *vs*. “MSYM” T1.

**Figure:**
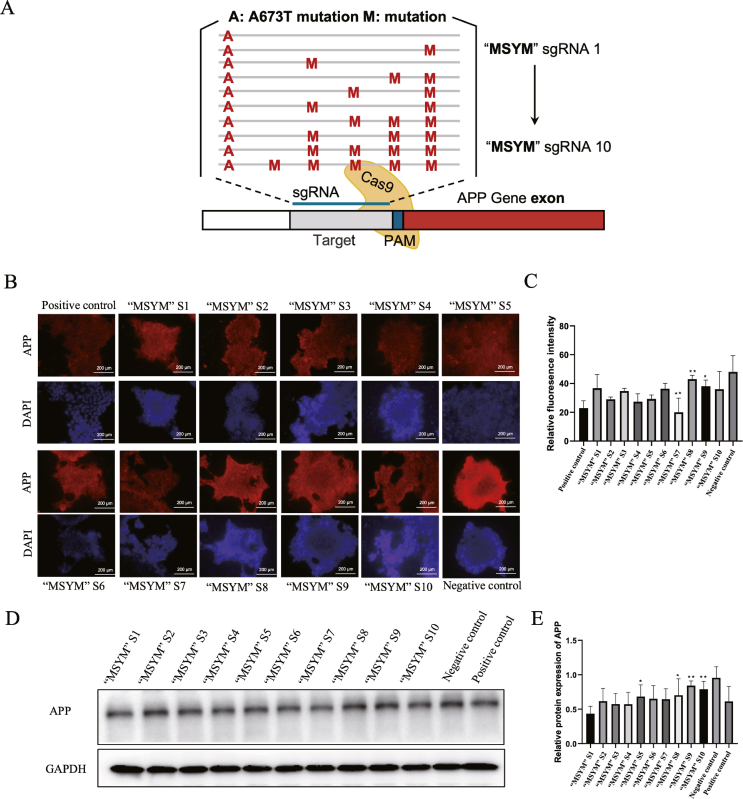
Figure S4 The cutting efficiency test using “MSYM” sgRNAs targeting APP gene. **(A)** “MSYM” sgRNAs were designed according to the sequence of MSYM templates. **(B, C)** HEK293T cells transfected with “MSYM” sgRNAs-pCas9-EGFP were labeled with anti-APP antibodies (red). Cellular nuclei were stained with 4′,6-diamidino-2-phenylindole (DAPI) (blue). Scale bars: 200 μm. **(D, E)** Protein levels of APP in HEK293T cells transfected with “MSYM” sgRNAs-pCas9-EGFP were detected by western blot analysis. All data are presented as mean ± standard error of the mean. ∗*P* < 0.05, ∗∗*P* < 0.01, ∗∗∗*P* < 0.001 *vs*. “MSYM” S1.

**Figure:**
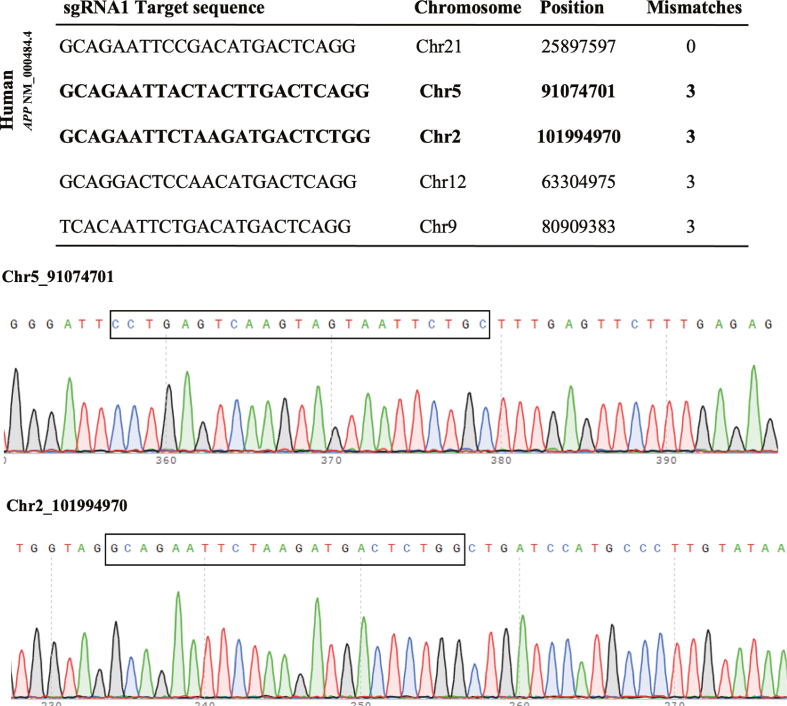
Figure S5 Off-targeting analysis was used to verify the A673T editing accuracy in iPS cells using MSYM.
