# Supplementary material for: Precise and efficient insertion of A673T mutation in APP gene using MSYM
Source: Genes Dis. 2023 Oct 27;11(6):101154. doi: 10.1016/j.gendis.2023.101154 (PMC11320440; doi:10.1016/j.gendis.2023.101154)
Supplement: Multimedia component 1 [file mmc1.docx]

**Materials and methods**

**Plasmid design and construction**

Single guide RNAs (sgRNAs) targeting the same 5′ region of the APP gene were designed using the Zhang laboratory CRISPR design tool (<http://crispr.mit.edu>). sgRNA sequences were then cloned into Cas9/gRNA（puro-mCherry）vector.

**Design of ssODN repair templates**

The 50-bp ssODN repair templates were designed with homologous genomic flanking sequence centered around the predicted CRISPR/Cas9 cleavage site and containing A673T mutation and/or synonymous mutations according to the central dogma of molecular biology (Table S1).

**T7E1 assay**

The target fragment near the mutation site of APP A673T was amplified by PCR using appropriate primers (Supplementary Table S1). The T7EN1 cleavage assay was performed as described^40^. In brief,

the target fragment was amplified from the extracted DNA by PCR and purified by PCR purification kit (Axygen, AP-PRCR-50). The amplicon was denatured by heating and annealing to form heteroduplex DNA, treated with 5 units of T7 endonuclease 1 (New England Biological Laboratory) at 37 ℃ for 30 minutes, and then analyzed by 2.5% agarose gel electrophoresis.

**Assessment of gRNA-DNA binding activity**

According to sgRNA1 target sequence, we generated all possible sequences with synonymous mutations of WT or with A673T mutation using R. 383 sequences with synonymous mutations of WT (11 with 1 mutation, 47 with 2 mutations, 101 with 3 mutations, 119 with 4 mutations, 77 with 5 mutations, 25 with 6 mutations, 3 with 7 mutations) and 384 sequences with synonymous mutations of WT with A673T mutation (numbers including the A673T mutation, 1 with 1 mutation, 11 with 2 mutations, 47 with 3 mutations, 101 with 4 mutations, 119 with 5 mutations, 77 with 6 mutations, 25 with 7 mutations, 3 with 8 mutations) were obtained (Supplementary Table. 3-6).

To assess the binding activity between different gRNAs and their target sequences, we used CRISPR-off pipeline v1.1.2 with the default parameters, which provides an approximate free energy computation method for the Cas9–gRNA–DNA complex binding^41^. The energy computation consists of four parts: the energy of RNA–DNA hybridization, gRNA unfolding, DNA duplex and PAM sequence for correction. RNAfold from ViennaRNA v2.5.1 package is used for gRNA unfolding energy computation^42^. Higher CRISPR-off score represents a higher binding tendency. The CRISPR-off scores were calculated for binding of WT APP gRNA with synonymous mutated sequences of WT/A673T target sequence, and binding of WT APP target sequence with synonymous mutated sequences of WT and A673T APP and APP gRNAs. The sequences pairs with mismatches above 6 didn't obtain CRISPR-off score. The ggmsa package is used for result visualization^43^.

**Cell culture and transfection**

iPS cells were maintained in mTeSR1 medium (Stem Cell Technologies) and 100550A iPS cells were kept in Stemflex medium (Thermo-Fisher) at 37°C in a 5% CO_2_ incubator. iPS cells were seeded on 12-well plates. When approximately 70% confluent, iPS cells were co-transfected the MSYM templates (MSYM1, MSYM9) with sgRNA1-pCas9-EGFP plasmid (1:1) using Lipofectamine Stem reagent. HEK293T cells were maintained in DMEM with 10% FBS, 2 mM Glutamax and 100 U per ml penicillin and 0.1 mg per ml streptomycin (all Life Technologies) at 37 °C with 5% CO_2_. HEK293T cells were seeded on 6-well plates at 350,000 cells per ml. When approximately 70% confluent, HEK293T cells were co-transfected the MSYM templates (MSYM1, MSYM2, MSYM3, MSYM5, MSYM6, MSYM8, MSYM9 and MSYM10) with sgRNA1-pCas9-EGFP plasmid (1:1) using Lipo3000. Scr7 was added in medium 4 hrs before transfection and continued until 48 hrs after transfection. Puromycin was added in the first replenishment of medium until 72h after transfection.

**Off-targeting analysis**

Gene edited APP were tested for off-target editing events predicted for each sgRNA by the Zhang laboratory CRISPR design tool (http://crispr.mit.edu), which also considers insertions or deletions in the guide RNA target sequence. The top five non-overlapping predicted off-target sites for each sgRNA from each tool were used.

**Statistical analysis**

All statistic tests and graphs were performed as recommended by GraphPad Prism 7.0. We replicated all experiments at least three times and presented the data as the mean ± SD. The differences between groups were analyzed by Student’s t-test. P < 0.05 indicated statistical significance. Figures in this manuscript were created with BioRender.com.

**Data and materials availability statement**

All data associated with this study are present in the paper or the Supplementary Materials. Any other relevant data are available from the corresponding author upon reasonable request.

**Compliance and ethics** *The author(s) declare that they have no conflict of interest.*

**Acknowledgements** *This work was supported in part by grants from National Natural Science Foundation of China (81701078), Natural Science Foundation of Heilongjiang Province of China (Outstanding Youth Foundation, YQ2022H003), China postdoctoral science foundation (2016M600261, 2018T110317), Heilongjiang Postdoctoral Financial Assistance (LBH-Z15163), Heilongjiang Touyan Innovation Team Program for their support.*
