# Supplementary material for: Precise and efficient insertion of A673T mutation in APP gene using MSYM
Source: Genes Dis. 2023 Oct 27;11(6):101154. doi: 10.1016/j.gendis.2023.101154 (PMC11320440; doi:10.1016/j.gendis.2023.101154)
Supplement: Multimedia component 2 [file mmc2.docx]

**Table S1. The primers for sgRNA1 and sgRNA2.**

| Human APP sgRNA1 | Forward | GCAGAATTCCGACATGACTC |
| --- | --- | --- |
|  | Reverse | GAGTCATGTCGGAATTCTGC |
| Human APP sgRNA2 | Forward | GGAGATCTCTGAAGTGAAGA |
|  | Reverse | TCTTCACTTCAGAGATCTCC |
