# Supplementary material for: Precise and efficient insertion of A673T mutation in APP gene using MSYM
Source: Genes Dis. 2023 Oct 27;11(6):101154. doi: 10.1016/j.gendis.2023.101154 (PMC11320440; doi:10.1016/j.gendis.2023.101154)
Supplement: Multimedia component 7 [file mmc7.docx]

**Table S6. MSYM templates sequences.**

| MSYM1 | AATACAGGTTCTGGGTTGACAAATATCAAGACGGAGGAGATCTCTGAAGTGAAGATGGAT**ACAGAATTCCGACATGACTCAGG**ATATGAAGTTCATCATCAAAAATTGGTACGTAAAATAATTTACCTCTTTCCACTA |
| --- | --- |
| MSYM2 | AATACAGGTTCTGGGTTGACAAATATCAAGACGGAGGAGATCTCTGAAGTGAAGATGGAT**ACAGAATTCCGACATGATTCAGG**ATATGAAGTTCATCATCAAAAATTGGTACGTAAAATAATTTACCTCTTTCCACTA |
| MSYM3 | AATACAGGTTCTGGGTTGACAAATATCAAGACGGAGGAGATCTCTGAAGTGAAGATGGAT**ACAGAATTTCGACATGACTCAGG**ATATGAAGTTCATCATCAAAAATTGGTACGTAAAATAATTTACCTCTTTCCACTA |
| MSYM4 | AATACAGGTTCTGGGTTGACAAATATCAAGACGGAGGAGATCTCTGAAGTGAAGATGGAT**ACAGAATTCCGACACGATTCAGG**ATATGAAGTTCATCATCAAAAATTGGTACGTAAAATAATTTACCTCTTTCCACTA |
| MSYM5 | AATACAGGTTCTGGGTTGACAAATATCAAGACGGAGGAGATCTCTGAAGTGAAGATGGAT**ACAGAATTCCGCCATGATTCAGG**ATATGAAGTTCATCATCAAAAATTGGTACGTAAAATAATTTACCTCTTTCCACTA |
| MSYM6 | AATACAGGTTCTGGGTTGACAAATATCAAGACGGAGGAGATCTCTGAAGTGAAGATGGAT**ACAGAATTTCGACATGATTCAGG**ATATGAAGTTCATCATCAAAAATTGGTACGTAAAATAATTTACCTCTTTCCACTA |
| MSYM7 | AATACAGGTTCTGGGTTGACAAATATCAAGACGGAGGAGATCTCTGAAGTGAAGATGGAT**ACAGAATTCCGCCACGATTCAGG**ATATGAAGTTCATCATCAAAAATTGGTACGTAAAATAATTTACCTCTTTCCACTA |
| MSYM8 | AATACAGGTTCTGGGTTGACAAATATCAAGACGGAGGAGATCTCTGAAGTGAAGATGGAT**ACAGAATTTCGACACGATTCAGG**ATATGAAGTTCATCATCAAAAATTGGTACGTAAAATAATTTACCTCTTTCCACTA |
| MSYM9 | AATACAGGTTCTGGGTTGACAAATATCAAGACGGAGGAGATCTCTGAAGTGAAGATGGATAT**ACAGAATTTCGCCACGATTCAGG**ATATGAAGTTCATCATCAAAAATTGGTACGTAAAATAATTTACCTCTTTCCACTA |
| MSYM10 | AATACAGGTTCTGGGTTGACAAATATCAAGACGGAGGAGATCTCTGAAGTGAAGATGGAT**ACAGAGTTTCGCCACGATTCAGG**ATATGAAGTTCATCATCAAAAATTGGTACGTAAAATAATTTACCTCTTTCCACTA |
